# Early Re-Do Surgery for Glioblastoma Is a Feasible and Safe Strategy to Achieve Complete Resection of Enhancing Tumor

**DOI:** 10.1371/journal.pone.0079846

**Published:** 2013-11-13

**Authors:** Philippe Schucht, Michael Murek, Astrid Jilch, Kathleen Seidel, Ekkehard Hewer, Roland Wiest, Andreas Raabe, Jürgen Beck

**Affiliations:** 1 Department of Neurosurgery, University Hospital Bern, Bern, Switzerland; 2 Institute for Diagnostic and Interventional Neuroradiology, University Hospital Bern, Bern, Switzerland; 3 Department of Neuropathology, University Hospital Bern, Bern, Switzerland; The Ohio State University Medical Center, United States of America

## Abstract

**Background:**

Complete resection of enhancing tumor as assessed by early (<72 hours) postoperative MRI is regarded as the optimal result in glioblastoma surgery. As yet, there is no consensus on standard procedure if post-operative imaging reveals unintended tumor remnants.

**Objective:**

The current study evaluated the feasibility and safety of an early re-do surgery aimed at completing resections with the aid of 5-ALA fluorescence and neuronavigation after detection of enhancing tumor remnants on post-operative MRI.

**Methods:**

From October 2008 to October 2012 a single center institutional protocol offered a second surgery within one week to patients with unintentional incomplete glioblastoma resection. We report on the feasibility of the use 5-ALA fluorescence guidance, the extent of resection (EOR) rates and complications of early re-do surgery.

**Results:**

Nine of 151 patients (6%) with glioblastoma resections had an unintentional tumor remnant with a volume >0.175 cm^3^. 5-ALA guided re-do surgery completed the resection (CRET) in all patients without causing neurological deficits, infections or other complications. Patients who underwent a re-do surgery remained hospitalized between surgeries, resulting in a mean length of hospital stay of 11 days (range 7-15), compared to 9 days for single surgery (range 3-23; p=0.147).

**Conclusion:**

Our early re-do protocol led to complete resection of all enhancing tumor in all cases without any new neurological deficits and thus provides a similar oncological result as intraoperative MRI (iMRI). The repeated use of 5-ALA induced fluorescence, used for identification of small remnants, remains highly sensitive and specific in the setting of re-do surgery. Early re-do surgery is a feasible and safe strategy to complete unintended subtotal resections.

## Introduction

The resection of enhancing tumor (gross total resection, GTR) is known to prolong progression free and overall survival [1,2]. The introduction of 5-amino levulinic acid (5-ALA) as an intra-operative tumor detection agent significantly increases the likelihood of achieving a GTR, which previously was only achieved in a minority of patients [3-6]. Furthermore, intra-operative electrophysiological function surveillance technologies for mapping and monitoring of motor, speech, and visual functions have helped to increase resection of glioblastoma adjacent to areas of presumed eloquence [7-10]. 

An early post-operative MRI (within 72 hours) is commonly used to verify whether a complete resection of enhancing tumor has been achieved [3]. Using MRI during surgery (intraoperative MRI, iMRI) allows assessment of the extent of resection prior to the end of anesthesia, and thus provides the possibility to restart tumor surgery and resect any detected tumor remnants [11-15]. A recent randomized controlled trial showed an increased rate of achieved GTR using iMRI compared to surgeries without iMRI (96% achieved GTR with iMRI versus 68% without iMRI, no 5-ALA used) [16], thus confirming the advantage of iMRI for improving the extent of resection in surgery for malignant glioma.

iMRI differs from postoperative MRI not only in the timing of imaging (during surgery versus after surgery), but mainly in the consequences drawn from image findings; tumor remnants visualized through iMRI may lead to further resection if located in ineloquent locations, whereas small remnants seen on postoperative MRI seldom lead to a second surgery with immediate resection [1,16]. 

In the absence of iMRI our institution follows a protocol in which patients with contrast enhancing tumor remnants in presumed non-eloquent locations are offered an early re-operation (re-do surgery) in order to obtain the optimal surgical result. We used neuronavigation for intraoperative guidance and 5-ALA for identification of the tumor remnant that eluded resection in the initial surgery. This report describes the burden for the patient, neurological deficits and EOR, as well as the value of 5-ALA and neuronavigation in the setting of early re-do surgery.

## Materials and Methods

### Patients

All patients operated upon between October 2008 and October 2012 were retrospectively screened for study inclusion. Inclusion criteria were (i) initially CRET eligible tumor, (ii) attempted CRET at the Department of Neurosurgery, Bern University Hospital, Switzerland, at initial surgery, (iii) diagnosis of glioblastoma based on histology and (iv) a CRET eligible tumor remnant >0.175 cm^3^ according to postoperative MRI. Demographics and presenting symptoms were retrieved from our prospectively managed tumor database as well as operative and hospital records. All patients were assessed neurologically based on National Institutes of Health Stroke Scale (NIHSS) on the day before surgery, and at 24 hours, 48 hours, and 3 months after surgery. New postoperative neurological deficits were regarded as permanent if they persisted at 3 months. The study was approved by the local Ethics committee (Kantonale Ethikkommission Bern; registration number 14-11-12). The Kantonale Ethikkommission Bern waived the need for a patient informed consent because the retrospectively-collected data was analyzed anonymously.

### Preoperative Imaging

All pre- and postoperative MRIs were performed on a 1.5- or a 3-T scanner with a head coil. Unenhanced and enhanced (magnetization-prepared rapid-acquisition gradient echocardiography sequences with 0.1 mmol/kg body weight gadolinium-DTPA given intravenously) T1 sequences without gap were obtained (256 x 256 matrix, rectangular field of view, 1-mm slice thickness). 

### Surgery, 5-ALA and intra-operative mapping and monitoring

Surgery was performed as previously published [9]. In brief, neuronavigation (Brainlab) and 5-ALA were used per protocol. Neuronavigation was used for planning, choosing the trajectory and initial guidance during surgery. Resection was completed according to 5-ALA-induced fluorescence. Intra-operative monitoring and mapping was used depending on the presumed eloquency of the tissue adjacent to the tumor, as previously published [8]. 

### Postoperative imaging and early re-do surgery

Postoperative MRI was scrutinized for contrast enhancing tumor remnants. Volumetric analysis through manual segmentation of the contrast enhancing tumor remnant was performed across all slices (VectorVision; Brainlab, Heimstetten, Germany) [9]. Patients with resectable tumor remnant >0.175 cm^3^ were informed of the potential benefit of a complete resection of enhancing tumor (CRET) and were offered a second intervention within a few days (re-do surgery). 

Peri-operative antibiotic prophylaxis was extended from single shot Cefuroxim 1.5g iv for initial surgery to Cefuroxim 1.5g iv every 8 hours for re-do surgery.

**Figure 1:**
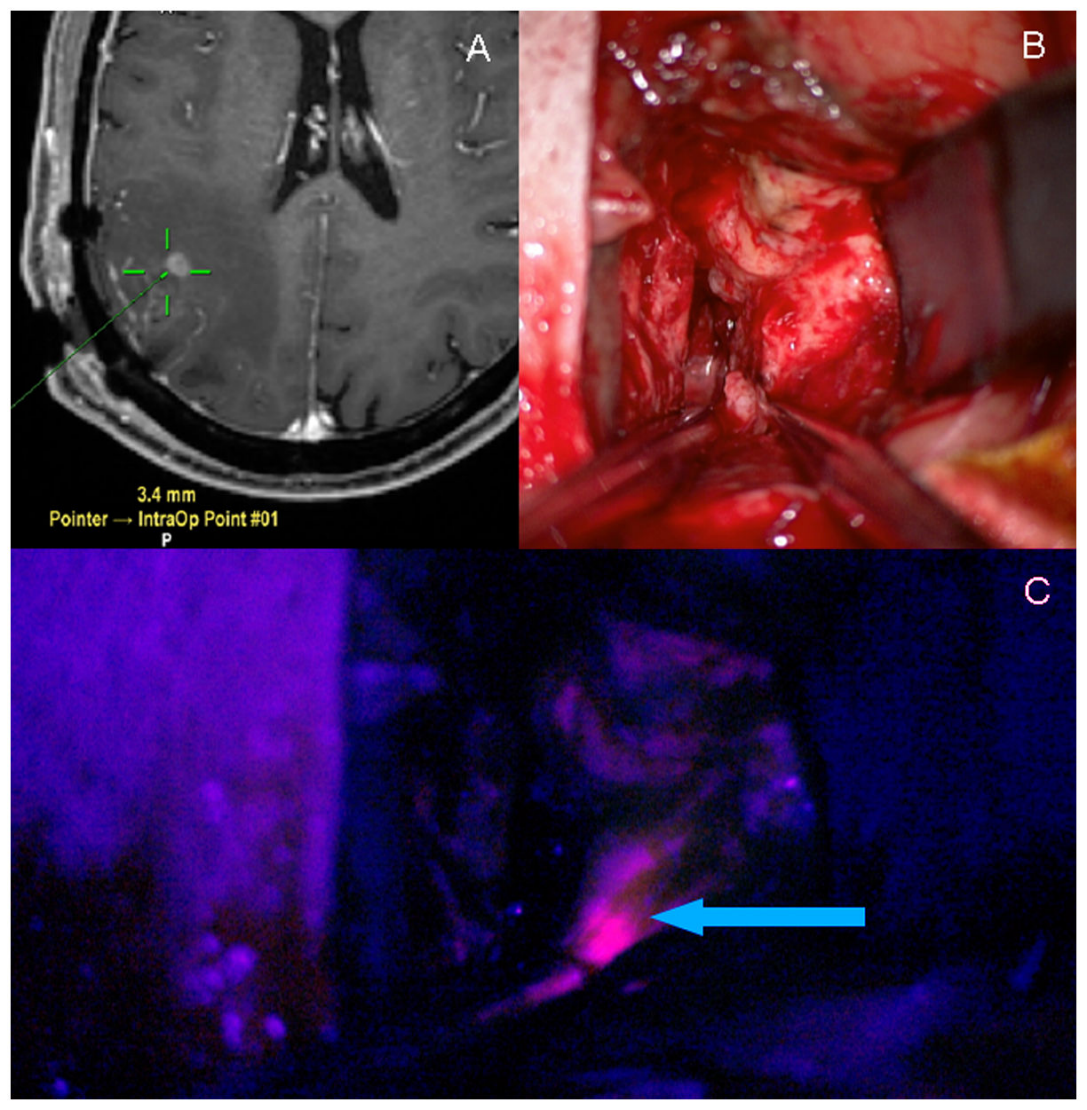
Navigation guides the approach and 5-ALA fluorescence identifies tumor remnant during re-do surgery. (A) Intraoperative neuronavigation guides the surgical approach towards the contrast enhancing tumor remnant (green crosslines = pointer navigation). (B) White light inspection of the surgical cavity. (C) 5-ALA induced fluorescence reveals tumor remnant under blue light (arrow).

Re-do surgery was performed using the same protocol as the first surgery. Neuronavigation was used to guide the surgical approach towards the small tumor remnant. Tumor remnant was identified through 5-ALA-induced fluorescence under blue light, resected and sent for histological confirmation (Figure 1). The postoperative MRI after second surgery was analyzed with consideration of the elapsed time after initial surgery; only the site of tumor remnant was investigated for remnants in order not to confuse remnants with postoperative inflammation [3]. Volumetric analysis of tumor remnant after re-do surgery was identical to the analysis after initial surgery. A re-do resection was judged CRET if the enhancing tumor remnant after initial surgery had vanished on post-operative MRI.

### Follow up examination

All patients were assessed using the NIHSS prior to discharge after the second surgery and again at a 3-month follow-up consultation. New postoperative neurological deficits were regarded as permanent if they persisted at 3 months. Hospital records were scrutinized for perioperative complications. In addition, patients were interviewed at follow-up regarding the occurrence of peri-operative complications.

## Results

### Initial Surgery

A total of 208 glioblastomas were operated upon from October 2008 to October 2012. Enhancing tumor tissue remnant with a volume of >0.175 cm^3^ that was left unintentionally was detected in the postoperative MRI of 9 patients (Table 1). Histological analysis was obtained in 7 patients. Solid or infiltrating tumor tissue was confirmed in 2 cases (patients 2 and 4) and 5 cases (#1, 3, 5, 8 and 9), respectively. Of these, 3 patients were female and the mean age was 63 years (range 38 - 78 years; Table 1). Neuronavigation had been used in all cases, 5-ALA in all but 1 case, and intra-operative mapping (IOM) had been used in 5 of the 9 patients. All tumors affected only one lobe: the frontal lobe in 4, the parietal lobe in 3 and the temporal lobe in 2 patients. The tumor was located on the right side in 7 patients. In 5 of the 9 patients the contrast enhancing tumor was within 10 mm of a presumed eloquent structure according to pre-operative MRI (CST in 4 patients and arcuate fascicle in 1 patient). One patient with a tumor near a presumed speech area was operated upon while awake to allow speech mapping. At the end of the surgery in all cases, the surgeon was certain that a complete resection had been achieved. Post-operative MRI was performed on the first day after surgery in all patients. A comparison to the initial pre-operative MRI revealed that the tumor remnants were not new lesions but in fact part of the initial tumor. Mean tumor volumes before and after first surgery were 24.3 cc (range 1.6 - 48.7 cc) and 1.0 cc (range 0.2-4.9 cc), respectively. The mean EOR of the first surgery ranged from 39% to 99% (median 98%). 

**Table 1:** Demographics and specifications of initial and re-do surgeries.

**Demographics**			**1st Surgery**						**Re-do Surgery**				
**Patient no.**	**Age (years)**	**Sex**	**Locali-zation**	**Initial volume (cm^3^)**	**Duration of surgery (min)**	**Intra-operative monitoring**	**Residual volume (cm^3^)**	**EOR (%)**	**Days between surgeries**	**Duration of surgery (minutes)**	**Intraoperative monitoring**	**EOR (%)**	**Duration of stay (days)**
1	76	f	Temporal Right	10.69	282	none	0.22	98	4	190	none	100%	14
2	61	m	Parietal Right	19.12	150	none	4.92	74	5	60	none	100%	15
3	73	m	Temporal Right	39.42	342	none	0.28	99	3	135	none	100%	8
4	58	f	Frontal Right	1.58	78	none	0.97	39	2	120	none	100%	9
5	65	m	Frontal Left	19.16	453	MEP, Speech (awake) and motor mapping	0.53	97	6	78	MEP, SSEP	100%	10
6	38	m	Frontal Right	12.91	211	MEP, SSEP, motor mapping	0.30	98	7	100	MEP, SSEP	100%	15
7	78	m	Parietal Right	48.66	177	MEP, SSEP	0.28	99	6	126	MEP, SSEP	100%	11
8	50	f	Frontal Right	44.13	315	MEP, motor mapping	0.89	98	2	81	MEP, SSEP	100%	7
9	67	m	Parietal Left	22.68	186	none	0.62	89	2	107	none	100%	14
Mean	63			24.26	244		1.00	98 (median)	4	111		100%	11
Range	38-78			1.58-48.66	78-453		0.22-4.92	38.8-99.4	2-7	78-190		100%	7-15

EOR, extent of resection; f, female; m, male; MEP, Maximum evoked potential; SSEP, Somatosensory evoked potential

One patient had a left-sided neglect after the first surgery, which persisted at the 3-month follow-up consultation. In all remaining patients, we observed no new neurological deficits after initial surgery and re-do procedure, nor at the 3-months post-operative control. No patients had a record of post-operative pneumonia, deep vein thrombosis, pulmonary embolism, hepatic failure, wound infections, skin healing issues or other medical problems.

### Re-do Surgery

Our offer to re-do surgery to optimize the surgical result was accepted by all 9 patients. Re-do surgery was scheduled and performed within 7 days after the first surgery (mean elapsed time: 4 days, range 2-7 days). Neuronavigation and 5-ALA were used in all re-do surgeries (Table 2). According to the surgeon neuronavigation was useful in all cases for guidance of surgery towards the tumor remnant. 5-ALA-induced red fluorescence revealed the tumor remnant in 7 cases; in one case 5-ALA fluorescence was weak and not useful according to the surgeon, and in one case fluorescence was not reported. A new, patchy pink fluorescence was observed in 2 patients (patients 5 and 7).

**Table 2:** Value of neuronavigation and 5-ALA in identifying tumor remnants.

**Patient no.**	**Value of Neuronavigation**	**Value of 5-ALA**	**Histology**	**Reason for incomplete 1st resection**
1	Guided surgery	Identified remnant	Infiltrating tumor	Not specified
2	Guided surgery	Not specified	Solid tumor	Remnant hidden by unsuspicious parenchyma
3	Guided surgery and identified tumor	Minimal; fluorescence was not specific	Infiltrating tumor	Not specified
4	Guided surgery	Identified remnant	Solid tumor	Remnant hidden by 5-ALA negative parenchyma
5	Guided surgery	Identified remnant and new unspecific fluorescence in resection cavity	Infiltrating tumor	Not specified
6	Guided surgery	Identified remnant	No histology obtained	Remnant hidden by 5-ALA negative parenchyma
7	Guided surgery	Identified remnant and new unspecific fluorescence in resection cavity	No histology obtained	Remnant hidden by 5-ALA negative parenchyma
8	Guided surgery	Identified remnant	Infiltrating tumor	Remnant hidden by 5-ALA negative parenchyma
9	Guided surgery	Identified remnant	Infiltrating tumor	Not specified

The second surgery was significantly shorter than the first surgery (mean 244 minutes for initial surgery [SD 115] versus 111 minutes [SD 39] for redo surgery, p ≤0.005, Student`s t-test). 

Re-do surgeries were generally performed by the same surgeon that performed the first surgery. In 4 cases the tumor remnant was found behind a 5-ALA negative parenchyma bridge during re-do surgery (Table 2). In the patient who had initially been operated upon without 5-ALA (patient 2), unsuspicious parenchyma separated the tumor remnant from the cavity. No specific reason for missing the 5-ALA positive tissue was determined in the remaining 4 cases.

Patients remained hospitalized between surgeries. The mean durations of hospital stays among the 9 patients undergoing re-do surgery and the 142 patients undergoing a single resection were 11 days (range 7-15) and 9 days (range 3-23; p=0.147) respectively. We observed no elevated liver enzymes after the first or second administration of 5-ALA. No new neurologic deficits were observed in the 9 patients at discharge and at the 3-month follow-up consultation, except for one patient (patient 7) who experienced a persisting left-sided neglect after the first surgery.

### Complications

One patient had a left-sided neglect after the first surgery, which persisted at the 3-month follow-up consultation. In all remaining patients, we observed no new neurological deficits after initial surgery and re-do procedure, nor at the 3-months post-operative control. No patients had any record of post-operative pneumonia, deep vein thrombosis, pulmonary embolism, hepatic failure, wound infection, skin healing issues or other medical problems.

## Discussion

Landmark publications in recent years provided convincing evidence that the complete removal of enhancing tumor produces the optimal surgical result for patients suffering from glioblastoma [1,2,16,17]. The scientific focus has thus shifted from the importance of complete resection towards the value of technologies and surgical strategies that aid achievement of complete resections. A recent randomized controlled trial on intraoperative MRI (iMRI) for glioblastoma surgery showed not only an increased rate of achieved gross total resections, but also indicated that patients who underwent a complete tumor resection might have a longer progression-free survival than patients with residual tumor [16]. Our protocol with early re-do surgery for unintended tumor remnants resembles the mentioned iMRI protocol in its ambition to provide complete resection; its main difference is the timing of imaging and remnant surgery. 

### Combined use of 5-ALA and neuronavigation for early re-do surgery

**Figure 2:**
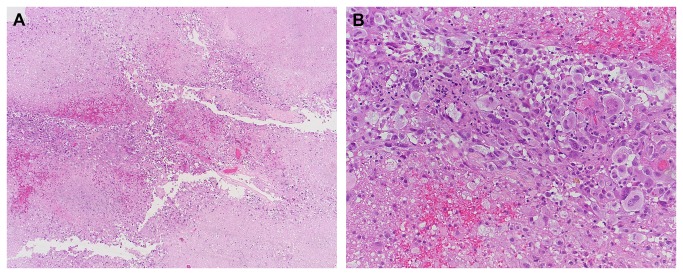
Histological analysis of tumor remnant resected during re-do surgery in Patient #8. (A) Areas of viable tumor (center) as well as radiation-type necrosis. (B) At higher magnification, pleomorphic astrocytic tumor cells – some of them with bizarre nuclear atypia – are seen. Scale bar corresponds to 400 micrometer (A) and 50 micrometer (B).

Detection of tumor remnants during re-do surgery may be a challenge given the small size of the remnants (mean 1 cc) and the fact that the remnants were overlooked during the first surgery. A combination of neuronavigation and 5-ALA-fluorescence was therefore applied in our series to reliably detect and resect tumor remnants. There are no reports on using 5-ALA for early re-do surgery to our knowledge, and 5-ALA should thus be used with caution. 5-ALA has a plasma half-life of less than 1 hour and cannot be detected after >6 hours in healthy brain tissue. However, the pharmacokinetics of 5-ALA in glioblastoma infiltrated tissue are not yet entirely understood, and prolonged leakage of 5-ALA from residual tumor cells may decrease 5-ALA’s specificity [18,19]. Furthermore, despite its high specificity for glioma tissue [20] there have been sporadic reports of non-specific, false positive fluorescence probably caused by inflammation [21-23]. This is of particular concern in the early post-operative setting as reparative processes lead to local edema and inflammation. Non-specific inflammatory processes at the resection border may also lead to a partial breakdown of the blood brain barrier as visualized by non-specific contrast enhancement on post-operative MRI performed >72 hours after surgery [3]. In two of our patients (#5 and #7, both re-operated after 6 days) a new pink fluorescence was detected in the resection cavity in addition to the bright red fluorescence of the tumor remnant under blue light. We believe that the interval between surgeries (6 days) was too short for actual tumor recurrence, and thus suspect that this new fluorescence is non-specific. Hence, we believe that 5-ALA must be used with caution in early re-do surgery and the possibility of unspecific fluorescence should always be considered. Combining 5-ALA with neuronavigation helped to verify suspected tumor remnants in our series and to rule out false positive fluorescence; histological analysis revealed solid or infiltrating tumor in all included cases (Table 2, Figure 2). Therefore, despite a decreased specificity, 5-ALA retained its value for intraoperative tumor identification even in those cases where new unspecific fluorescence was observed.

### Infections and Complications

Infections are a major concern after re-operations. Performing a second surgery within a few days entails re-opening the skin prior to proper healing, which may facilitate infections. While there are no reports on infections after early re-do surgery for glioma, there are data on infections for resection of recurrent glioma. The Glioma Outcome Project reported an increased rate of general infections after surgery for recurrent glioblastoma (4.4%, compared to 0% after first surgery, p<0.0001) [24], according to the authors possibly due to prolonged use of corticosteroids. The reported rates of local infections, however, were similar after craniotomy for initial and recurrent tumor [24]. The absence of infections in our series is thus in line with the existing literature.

Similarly, our concern that the physical stress of a second surgery within a short time period might lead to a higher rate of medical complications turned out to be unfounded for this series, as we observed no medical complications and no wound healing problems in the peri-operative period up to 3 months after surgery. 

The small sample size of our study does not allow an extrapolated analysis of infections and complications. Larger studies are warranted to assess the true rate of infections and complications for two consecutive surgeries within a short time interval as described in this study.

### Burden of re-do surgery compared to iMRI

Our protocol with early re-do surgery requires a second anesthesia and may increase patients’ physical and psychological stress. However, this additional stress was only necessary in a minority of our patients (9 out of 208 patients, 4.3%). This contrasts with the use of iMRI; in a recent randomized controlled trial iMRI led to further resection in one-third of all patients [16]. Thus, anesthesia was prolonged without apparent benefit to two-thirds of all patients (429 min for iMRI vs 362 min in conventional group, p=0.007) [16]. Of note, 5-ALA was not used in the mentioned computed tomography on iMRI but is administered per protocol to all patients with presumed glioblastoma at our institution. 5-ALA’s ability to increase the likelihood of complete resection may explain the higher rate of incomplete resection before control MRI in the computed tomography on iMRI as compared to our series. The ‘number to scan’ to achieve surgical improvement through iMRI in a single patient may well be higher when using 5-ALA. A protocol of early re-do surgery thus leads to a greater burden for a small percentage of patients, while routine iMRI imposes a small additional burden on all patients.

## Conclusion

Re-thinking the efforts to achieve a complete resection of enhancing tumor is warranted in the wake of the 5-ALA study group`s assessment of the effect of GTR on survival. Providing glioblastoma patients with a MRI-controlled optimal surgical result should not be a privilege of centers with intra-operative MRI. In the absence of iMRI, our institutional protocol calls for early re-do surgeries (within a week) for unintentional incomplete resection as detected by early postoperative MRI. Re-do surgery achieved a complete resection (CRET) in all patients without leading to additional neurological deficits. A combination of neuronavigation for surgical guidance and 5-ALA for tumor identification enabled reliable detection and removal of tumor remnants in all cases. For neurosurgical centers that cannot provide iMRI, early re-do surgery may thus be a feasible strategy to increase the rate of complete resections in glioblastoma patients.
